# Consumers stated and revealed preferences for community health workers and other strategies for the provision of timely and appropriate treatment of malaria in southeast Nigeria

**DOI:** 10.1186/1475-2875-5-117

**Published:** 2006-12-01

**Authors:** Obinna Onwujekwe, Nkem Dike, Juliana Ojukwu, Benjamin Uzochukwu, Nkoli Ezumah, Elvis Shu, Paul Okonkwo

**Affiliations:** 1 Health Policy Research Group, Department of Pharmacology and Therapeutics, College of Medicine, University of Nigeria, Enugu, Nigeria; 2 Department of Paediatrics, Ebonyi State University Teaching Hospital, Abakalikin, Nigeria; 3 Department of Community Medicine, College of Medicine, University of Nigeria, Enugu, Nigeria; 4 Department of Sociology and Anthropology, University of Nigeria, Nsukka, Nigeria

## Abstract

**Background:**

The African Heads of State meeting in Abuja, Nigeria on Roll Back Malaria adopted effective treatment of malaria nearer the home as one of the strategies for malaria control in Africa. A potentially effective strategy for bringing early, appropriate and low cost treatment of malaria closer to the home is through the use of community health workers (CHWs). There is paucity of information about people's actual preferences for CHWs and how stated preferences relates to revealed preferences for both the CHW strategy and other strategies for improving the timeliness of malaria treatment in not only Nigeria but in many malaria endemic countries.

**Objectives:**

To determine peoples' stated and actual preferences for different strategies for improving the timeliness and appropriateness of treatment of malaria before and after the implementation of a community health workers (CHW) strategy in their community.

**Methods:**

A prospective study was undertaken in a rural malaria holo-endemic Nigerian community. A questionnaire was used to collect information on health-seeking from householders before (first survey) and after (second survey) implementation of a CHW malaria treatement strategy.

**Results:**

The consumers mostly preferred the CHW strategy over self-treatment in the homes and other strategies of treatment. The use of community health workers (CHWs) increased from 0% to 26.1% (p < 0.05), while self-treatment in the homes decreased from 9.4% to 0% (p < 0.05) after the implementation of the CHW strategy. Use of patent medicine dealers also decreased from 44.8% to 17.9% (p < 0.05) after CHW strategy was implemented.

**Conclusion:**

Community health workers can be used to improve and ensure timely and appropriate treatment of malaria. The CHW strategy could also be sustained since it was preferred and used by consumers over self-treatment in the homes as well as other strategies for improving treatment. Hence, the CHW strategy is a feasible and promising method of improving home-management of uncomplicated malaria.

## Background

The African Heads of State meeting in Abuja, Nigeria on Roll Back Malaria adopted effective treatment of malaria nearer the home as one of the strategies for malaria control in Africa [1]. In most part of sub-Saharan Africa (SSA), the public health infrastructure is inadequate to fully meet existing needs [2-4]. When also viewed from the angle that anti-malarial drug resistance is forcing newly developed and often times expensive drugs such as artemisinin-based combination therapy into scaled-up accelerated use, treatment strategies that would ensure that such drugs are appropriately and timely used would require innovative approaches [3]. Although large numbers of sick children have no contact with health facilities, few countries have made quality care for malaria or pneumonia available as scale outside of health facilities [5].

A potentially effective strategy for bringing early, appropriate and low cost treatment of malaria closer to the home is through the use of community health workers (CHWs). This could be within the framework of the home-management strategy currently being promoted by WHO in Africa or Integrated Management of Childhood Illnesses (IMCI) [5-7]. The CHWs would be better able than most householders to recognise the symptoms of malaria, prescribe/dispense appropriate medication, ensure compliance to treatment and provide a reliable referral point when treatment fails and for complicated malaria cases [8]. CHWs may serve to increase the coverage and equity of service delivery at low cost [9,10]. According to Kahssay [11], the question of whether CHWs serve to improve health for all is no longer relevant; rather the question is how their potential can be realized. There is a growing demand for CHWs to take on not only the management of malaria, but also diarrhoea treatment with zinc and oral re-hydration salts [5].

There is paucity of information about people's actual preferences for CHWs and how stated preferences relates to revealed preferences for both the CHW strategy and other strategies for improving the timeliness of malaria treatment in not only Nigeria but in many malaria endemic countries. Nonetheless, health service delivery programs using minimally-trained CHWs have increasingly been established in many countries both in large-scale and small-scale with varying goals and degrees of success [9,10]. Depending on where CHWs have been established or are still being introduced, from the USA [9,12], to Asia [13-15], to Africa [16-20], the umbrella term "community health worker" encompasses a variety of health assistants who are selected, trained and work in the communities in which they live. CHWs are referred to by various names such as village health workers, volunteer/voluntary health workers, community resource persons, village health volunteers and village-based volunteer workers.

Community health workers have played an important role in malaria diagnosis and treatment in many different settings for more than 35 years [5,14,15,19,21-24]. The use of CHWs can increase the number of patients receiving anti-malarial drugs [5,19,23] and can increase the correct administration of drug regimens in the home [5]. In The Gambia, Ethiopia and Democratic Republic of Congo, use of CHWs led to reductions in malaria mortality [19,22,23].

However, despite this evidence of the benefits of improving access to treatment using CHWs, some experts remain cautious about this approach, because of concerns that allowing CHWs to distribute anti malarials will increase the misuse of drugs and accelerate the development of anti malarial resistance [25]. Also, despite the extensive body of research, few sustainable CHW programmes have been implemented to scale in Africa and CHW programmes usually fail because of high drop-out rates or weak commodity distribution systems. Hence, the CHW model may be un-scalable, but the experiences with the wide-scale use of community directed distributors (CDD) of ivermectin within the framework of community-directed treatment with ivermectin (CDTI) shows that the CHW strategy is feasible and could be scaled-up as long as it is properly developed using the preferences of the communities in programming and with active community involvement in all aspects of the intervention [26].

Hence, this paper compares stated and actual preferences for strategies for improving timely treatment of malaria with special emphasis on the use of the CHWs. Using peoples' preferences to drive policies and programmes is important in order to ensure sustainability of malaria and other disease control efforts [27]. The results provide evidence that the CHW strategy is an acceptable method for not only achieving timely treatment of malaria, but could also be used to improve the home-management of malaria. Seven CHW models for case management of children with malaria and/or pneumonia outside of health facilities, based on a systematic review of literature on the CHW strategy have been identified [5]. This study was mostly based on Model 3, which is CHW-directed fever management that entailed the provision of malaria treatment and verbal referral. However, there were slight elements of Model 4 because families in some cases influenced the choice of given to be given to the sick people due to their preferences and levels of ability to pay for the different drugs.

## Materials and methods

### Study area and design

The study was undertaken in two comparison and intervention villages respectively from Achi community, Oji-River local government area of Enugu State, southeast Nigeria. Achi is comprised of a projected population of 20,000 people, is holoendemic for malaria and *Plasmodium falciparum *causes more than 90% of all malaria cases there [28].

Achi has a total of 12 villages. Direct allocation with the aid of local malaria control team was used to select the comparison and intervention villages. The intervention site was two villages namely, Adu and Ahani while the comparison villages were Amaetiti and Enugwu-akwu. There is no functional community health worker for the treatment of malaria in Achi, although there were a number of community-directed distributors of ivermectin for the control of onchocerciasis. There is a public hospital, which is located in the comparison villages, whilst the health centres are located in the intervention villages. There is one private hospital and comparison and intervention villages respectively. There are a number of patent medicine stores in both the intervention and comparison villages and itinerant drug providers also visit Achi on the major market days. Numerous herbalists abound in the comparison and intervention villages.

The study was conducted in three phases: (1) the first survey that was used to collect baseline data in both the comparison and the intervention villages; (2) the implementation of the CHW strategy in the intervention villages; and (3) a second survey to evaluate the intervention at the end of the second phase, which was conducted in both the intervention and the comparison villages.

### The surveys

Two cross-sectional surveys were conducted six months apart (March and September) so as to collect data on peoples' stated preferences for near and appropriate treatment of malaria as well as treatment sources before the introduction of the CHW strategy (1^st ^survey) and after the implementation of the CHW strategy (2^nd ^survey). Hence, it covered the periods when cash is in short supply during the planting season (March) to when cash is plentiful during and after the harvest season (September). Pre-tested structured questionnaires were used to collect the data from random sample of 600 respondents from the intervention and comparison villages respectively in the first survey and from a random sample of 300 respondents in the second survey. Households were randomly selected from the sample frame, which were the household lists in the villages. The decrease in the number of respondents in the second survey was due to limited resources needed to re-interview 600 people. The respondents were the heads of households or their representatives (where the head was absent).

Trained and educated interviewers who were resident in the villages administered the questionnaires to the respondents after obtaining their informed consent. In the first survey, the respondents were questionned about their health-seeking behaviour for the treatment of malaria as well as their preferences or strategies for improvement of timely and appropriate treatment. In the second survey, the respondents were asked similar questions as in the first survey, but in addition, the actual health care-seeking behaviour for malaria treatment were determined. Before asking the respondents about their preferences for improvement of malaria treatment, some strategies for improved timeliness and appropriateness of treatment of malaria were presented to them in the questionnaire. The strategies were self-treatment in the home, treatment by patent medicine dealers and shop-keepers, treatment in primary healthcare centers, education to mothers to self-treat, treatment by community-based health workers and others (treatment in hospitals, clinics etc). In examination of treatment-seeking, people were asked about their most recent episodes of malaria within one month to the date of the interview and they were asked where they sought treatment for their presumptive malaria.

### Implementation of the CHW strategy

Discussions at village assemblies with broad segment of community leaders and an interactive meeting with all the stakes-holders were used to fine-tune the design the CHW strategy in the intervention villages. The participants included staff the local government malaria control staff and community leaders. Important factors that were considered in the design were the criteria and method of selecting the CHWs, type of training to be given and type of services they should provide. Furthermore, the source of payment for the CHWs, their role in the referral mechanism for complicated cases and the role in the broad public health system were fashioned.

The study team trained selected community members to treat uncomplicated cases of presumptive malaria or fevers. There were seven CHWs in the intervention villages. Four of them were located in Ahani, while three of the CHWs were in Adu. The training lasted for one month. The CHWs were trained to treat only uncomplicated cases of fever and to refer all other cases to the health centre or general hospital within Achi town. Pregnant women were excluded from being treated by the CHWs. The CHWs were taught on how to verbally refer cases with persistent fever to the health centre or hospital that was nearest to the sick person. At the end of the training, the candidates were issued with identity cards, which were signed by the primary health care coordinator and then officially became CHWs. The CHWs were also supplied with drugs, stationary and other materials by the project team and they started performing their duty after thereafter. The CHWs were paid a commission on the drug sales. Therefore, a reasonable mark-up was addeded to all the drugs and this mark-up was paid to the CHWs at the end of every month. However, the prices for the drugs were not more than the available prices in the market.

The project team gave the CHWs treatment guidelines and two anti-malarial drugs namely chloroquine (syrup and tablets) and a sulfadoxine-pyrimethamine (SP) preparation in tablet form were used. These two drugs were the first-line drugs for the treatment of malaria in Nigeria then, until early 2005 when the first line drugs were officially changed to artemisinin-based combination therapy (ACT). However, because ACTs were not yet officially available, chloroquine and SP were still highly used to treat malaria. The strategy was implemented and monitored in the villages for 5 months and then evaluated. The CHWs had the discretion to prescribe any of the drugs, but the consumers preferences were allowed to guide the prescriptions.

People that had malaria or fever visited the CHWs homes for treatment, but people that could not go there were treated at their homes by the CHWs. The CHWs used treatment forms and registers to keep records of people that they treated. They also used cash registers to record their revenues and issued receipts to people that paid for their services. The CHWs kept their drug stocks in their homes, together with the thermometers and other materials. They sent the revenues they realize to either their supervisors or to the local project manager, who in turn send the money to the project director. The prices of the drugs depended on their costs. For SP, the cost of a full adult dose was used to grade the prices of the drug. A full adult dose cost 110 Naira (US$1.0) from the manufacturers and hence a maximum mark-up of 25 Naira (US$0.23) was added as the commission to the CHWs. For chloroquine, a full adult dose cost 31 Naira (US$0.28) from the manufacturers and hence a 19 Naira (US$0.17) mark-up was added. 20 Naira (US$0.18) mark-up was added to chloroquine and paracetamol syrups, 0.05 Naira (US$.0005) to paracetamol tablet and 30 Naira (US$0.28) to the blood tonic.

The CHWs administered questionnaires to all their patients for the collection of information of household socio-economic characteristics, the antimalarial they took and how they paid for treatment. The CHWs also used another form to collect clinical data about the patients. These were the symptoms and signs that the patients presented with, including their body temperature.

### Operational definitions that were used in this study

#### Self-treatment at home

Diagnosis and treatment of malaria cases with drugs kept in the homes at the time a householder has malaria without involvement of healthcare services.

#### Home management

In this case, the diagnosis, treatment, prescription of drugs could have been provided by healthcare providers, but the care of the patient until s/he recovered is undertaken in the home. Once the drug is procured outside the home on the advice of a health care provider when someone already has malaria, then the subject is not home treatment but home management. Home-management of illnesses equally applies to most disease conditions where either the ill or recuperating patients are cared for in their homes.

#### Community health workers

They encompass a variety of health assistants who are selected, trained and work in the communities in which they live.

#### Appropriate treatment

Treatment of presumptive malaria with adequate doses of correct anti-malarial drugs.

### Data analysis

The revealed preferences as well as actual preferences were compared before and after the implementation of the CHW strategy. In addition, the data from the two intervention villages were compared to that of the comparison villages. Also, within each village group, the stated preferences of the respondents for different treatment options before and after the implementation of the CHW strategy were compared. In addition, the sources of treatment (revealed preferences) before and after the implementation of the CHW strategy were compared between the intervention and comparison villages and within each village group. There were logistic regression analyses of stated and revealed preferences for CHWs so that factors that would explain them would be determined. The independent variables in the logistic regression analysis included data on socio-economic characteristics of the respondents and their households, whether they were resident in intervention or comparison villages as well as information on ownership of a radio, bicycle and motorcycle together with the per capita weekly value of food. A full-to-reduced modelling approach was used in order to arrive at the best regression models. The independent variables with the smallest t-statistic, and whose removal did not adversely affect the other coefficients nor the prediction of the models, were removed sequentially.

## Results

### Respondents' and households' characteristics

Household heads, females, married people and middle-aged people were the majority of the respondents in both the intervention and comparison villages (Table 1). Most of the respondents in the intervention villages had formal education, while the opposite held in the comparison villages. Majority of the households owned a radio set and bicycle, but not many households owned motorcycle or motorcar in both the intervention and comparison villages. The comparison and intervention villages were statistically similar except for education, and ownership of bicycle, motorcar and food value.

**Table 1:** Respondents'/household socio-economic and demographic characteristics

	Intervention villages N = 597 n (%)	Comparison villages N = 600 n (%)	Chi-square
Household head	352 (59.0)	373 (62.2)	1.28
Female	364 (61.0)	353 (58.8)	0.57
Age (years): Mean (SD)	54.04 (15.16)	53.52 (15.16)	0.24
Had formal education	344 (57.6)	255 (42.3)	27.34***
Married	531 (88.9)	528 (88.0)	0.26
No of household residents: Mean (SD)	4.51 (2.98)	4.32 (2.45)	0.70
Household owns a radio	495 (82.9)	464 (77.3)	5.85**
Household owns a bicycle	327 (54.8)	369 (61.5)	5.55**
Household owns a motorcycle	111 (18.6)	94 (15.7)	1.80
Household owns a motorcar	44 (7.4)	30 (5.0)	2.90
Average weekly per capita food value (SD)	368.41 (384.62)	299.14 (315.28)	16.18**

Note: * = p < .10; ** = p < .05; *** = p < .01

### Preferences for different providers before and after implementation of CHWs strategy

Treatment by CHWs was mostly preferred in the comparison and intervention villages as a means of providing near, timely and appropriate treatment of malaria, whilst treatment in health centres was the second preferred option (Table 2). Self-treatment in the home was the third preferred option in the intervention site before and after implementation of the CHW strategy. When compared to the preferences before the CHW strategy was started (Table 2), there was a marked increase in the proportion of people that preferred CHWs in intervention site, whilst it declined in the comparison site but this decline was not statistically significant. Conversely, the decline in the preference of self-treatment in the home was statistically significant in both intervention and comparison villages. Training of shopkeepers and mothers in order to improve their malaria treatment practices were the least preferred.

**Table 2:** Preferences for different providers for the provision of near and appropriate treatment before and after implementation of CHW strategy

**Before implementation of CHW**	Intervention villages N = 600 n (%)	Comparison villages N = 600 n (%)	Chi-square
Self-treatment at home	68 (11.3)	135 (22.5)	26.2***
Community health workers	315 (52.5)	283 (47.2)	3.74*
Education to mothers to self-treat	35 (5.8)	29 (4.8)	0.63
Treatment in primary health centers	72 (12.0)	82 (13.7)	0.69
Train patent medicine dealers (drug sellers)	28 (4.7)	2 (0.3)	23.14***
Hospitals	49 (8.2)	22 (3.7)	11.05***
**Others (including herbalists)**	**3 (0.5)**	**7 (1.2)**	1.59
**After implementation of CHW**	**N = 300**	**N = 300**	
Self-treatment at home	17 (5.7)	20 (6.7)	0.28
Communityd health workers	209 (69.7)	121 (40.3)	48.45***
Education to mothers to self-treat	9 (3.0)	31 (10.3)	13.0***
Treatment in primary health centers	41 (13.7)	75 (25.0)	10.7***
Train patent medicine dealers (drug sellers)	1 (0.3	40 (13.3)	40.04***
Hospitals	15 (5.0)	8 (2.7)	2.16
Others	8 (2.7)	2 (0.7)	3.70*

Note: * = p < .10; ** = p < .05; *** = p < .01

### Treatment-seeking (revealed preferences) before and after implementation of CHW strategy

Before the implementation of the CHW strategy, patent medicine dealers were the most common source of treatment in both the intervention site (44.8%) and comparison site (52.4%) (Table 3). A total of 9.4% and 7.1% of the people who had malaria used self-treatment at home in the intervention and comparison villages respectively. The differences in uses of different providers before the implementation of the intervention were only statistically significantly different for use of hospitals and "others". Table 3 also shows that both the comparison and intervention villages had similar malaria treatment-seeking behaviours with regards to all the sources of treatment with the exception of hospitals, which was used more by the intervention villages. However, after the implementation of the CHW strategy, the comparison and intervention villages differed in sources of treatment for all treatment sources with the exception of hospitals.

**Table 3:** Places where treatment was first sought before and after implementation of CHW strategy

**Before implementation of CHW**	**Intervention villages** **N = 212** **n (%)**	**Control villages** **N = 168** **n (%)**	**Chi-square**
Self-treatment at home	20 (9.4)	12 (7.1)	0.99
Community health worker	0 (0)	0 (0)	Not applicable
Treatment in primary health centers	20 (9.4)	25 (14.9)	1.87
Patent medicine dealer (drug sellers)	95 (44.8)	88 (52.4)	0.59
Hospital	44 (20.8)	17 (10.1)	9.37***
Others (including herbalists)	33 (15.6)	26 (15.5)	0.96
**After implementation of CHW**	**N = 84**	**N = 90**	
Self-treatment at home	0 (0)	6 (6.7)	5.72**
Community health worker	22 (26.1)	1 (1.1)	23.6***
Treatment in primary health centers	20 (23.9)	12 (13.3)	3.33*
Patent medicine dealer	15 (17.9)	52 (57.8)	3.17*
Hospital	7 (8.3)	3 (3.3)	2.01
Others (including herbalists)	20 (23.8)	16 (17.7)	0.54

Note: * = p < .10; ** = p < .05; *** = p < .01

After the implementation of the CHW strategy, there was a decline in use of patent medicine dealers and shopkeepers from 44.8% to 17.9% in the intervention site (p < 0.05), whilst it was more or less stable in the comparison site at 52.4% before and 57.8% after implementation (p > 0.10). Also, the use of self-treatment at home dropped to zero in the intervention site, whilst it remained static at 6.7% in the comparison site after the implementation of the CHW strategy. The use of CHWs increased to 26.1% from 0% in the intervention site whilst it increased to 1% in the intervention site.

The differences in stated preferences in both intervention and comparison villages before and after the implementation of the CHW strategy were statistically significant for all the options except for use of primary healthcare centres in intervention site and CHWs in the comparison site. Treatment-seeking (actual preferences) in the intervention site was statistically significantly different (p < 0.05) between all sources of treatment before and after the implementation of the CHW strategy. However, in the comparison site, it was only statistically significantly different for use of patent medicine dealers (p = 0.07).

### Logistic analyses

The location of the respondents were statistically significantly related to both preferences and use of CHWs in both the intervention and comparison villages before and after the implementation of the CHW strategy (p < 0.01) (Table 4). The use of CHWs changed from a negative association with location before the implementation to a positive association after the implementation of the strategy. Formal education, being a female, and ownership of a radio were all positively and statistically significantly associated with stated preferences for CHWs after the implementation. The result also shows that younger people, people with some formal education were more likely to use services of CHWs both before and after the implementation of the intervention. Also increasing number of household residents was positively associated with use of CHWs, while the ownership of bicycles was negatively associated with use of CHWs.

**Table 4:** Reduced logistic regression models on determinants of preferences and treatment-seeking from CHW

	Preferences for CHW before intervention Coeff. (SE)	Preferences for CHW after intervention Coeff. (SE)	Treatment-seeking for CHW before intervention Coeff. (SE)	Treatment-seeking for CHW after intervention Coeff. (SE)
Location	.19 (.12)***	1.48 (.19)***	-1.59 (.34)***	3.97 (1.35)***
Status in household	- - - - - -	- - - - - -	- - - - - - -	1.05 (.90)
No of household residents	.10 (.02)***	- - - - - -	-.09 (.07)	0.31 (.15)**
Gender	.29 (.13)**	-.45 (.19)**	- - - - - - - -	- - - - - -
Age	.005 (.004)	.007 (.006)	-.02 (.01)**	-.06 (.03)*
Had formal education	-.22 (.12)	.56 (.33)*	.60 (.33)*	1.55 (.87)*
Marital status	- - - - - - -	- - - - - -	-.76 (.41)	- - - - - - -
Had malaria	.33 (.16)***	- - - - - -	- - - - - - -	- - - - - - -
Weekly per capita food value	.001 (.0001)***	- - - - - -	-.002 (.001)***	.001 (.0008)
Owns a radio	- - - - - -	.36 (.21)*	-.59 (.35)*	- - - - - - - -
Owns a bicycle	- - - - - -	- - - - - -	- - - - - - - -	-1.7 (0.75)**
Owns a Motorcycle	- - - - - -	- - - - - -	- - - - - - - -	- - - - - - - -
Owns a motorcar	-.25 (.25)	- - - - - -	- - - - - - - -	1.90 (1.38)
Constant	-1.15 (.32)***	-1.39 (.51)***	2.02 (.80)**	-5.01 (2.03)**

LR Chi-square	54.0***	73.7***	73.2***	56.6***
Pseudo R2	0.03	0.10	0.19	0.45
Correct predictions	58.6%	67.2%	84.2%	90.6%

Note: * = p < .10; ** = p < .05; *** = p < .01

## Discussion

The stated preferences of the respondents for malaria treatment strategies were in most cases at variance with their revealed preferences, which imply what they do in real life. However, stated preferences were only determined for strategies to improve treatment, whilst actual preferences were for existing providers. Hence, although not directly comparable, the results provide good insights on differences between stated and revealed preferences. The training of patent medicine dealers and shop-keepers, though desirable because they are the major source of drugs in the community, were not highly preferred by the respondents possibly because people felt that the quality of services there was sub-optimal. Nonetheless, one could argue that people used the services of patent medicine dealers most before the implementation of the community health worker (CHW) strategy in intervention villages because they were available in large numbers and community health workers (CHWs) were non-existent in the villages.

The decrease in preferences and use of self-treatment at home and patent medicine dealers in the intervention site implies that in the presence of community-based alternative for providing timely and appropriate treatment of malaria, that strategy would be preferred by the people. However, it is possible that the preferences were not for CHWs per se, but for any new approach over maintaining the status quo. Nonetheless, these findings do not imply that CHWs strategy should be seen as the ultimate strategy for the treatment of malaria at the community level, but it should be seen as a means of supporting the formal public health sector and private sources of treatment where the quality should be made optimal. Any intervention to improve management of sick people at the community level should ideally be part of a larger package that includes improving quality of care at facilities and improvements of health systems [5]. The fact that newer anti-malarials such as artemisinin-based combination therapy (ACT) are high cost, have complex regimens coupled with concern about resistance and poor diagnosis requires a new vision of malaria treatment policy and practical and substantial investments into public and private sectors' health infrastructure [3].

Despite the findings, the study is limited by the fact that only two villages in each instance within the same community were used for the intervention and comparison respectively. Whilst this had the advantage of ensuring that the comparison and intervention villages were comparable, it however limited the number of samples that could be used for the comparison, which could have provided more programmatic information for scaling-up the intervention. However, the intensive nature of the approach, as implemented in this study, could limit its scaling-up and an investigation of stated preferences for a more stripped-down and potentially more easily scalable CHW strategy would have shown whether preferences will still be the same.

Furthermore, there could have been intra-cluster correlation at the level of the CHW as one or two high performing CHWs could easily account for the entire observed effect of the intervention. However, because the data collection process was not structured to detect the effect of the individual seven (7) CHWs, but rather to detect community effects, it is not possible to analyse for possible intra-cluster correlation. Also, the fact that fewer respondents were re-interviewed after the implementation of the intervention could have limited the information that were elicited. Qualitative studies could have helped to gain more insights about why certain preferences were made and changed, as well as how to take the CHW strategy to scale. These limitations were occasioned by the limited resources available for the study but, these limitations do not overtly diminish the addition to knowledge of these findings and inferences.

Nonetheless, the implication of the findings is that the villagers do not really feel very confident to take on the added responsibility of self-treatment at home, if they have comparable or better alternatives for treatment. They would prefer that knowledgeable community members are trained to provide them with near and appropriate treatment of malaria. However, in the mid- to long-term basis, strategies to address the improvements in public health sector should address the specific reasons why people might not use the services there such as distance to facilities, lengthy waiting time, costs, lack of drugs [3,29-31]. This is because a rejuvenated properly functioning public healthcare system offers the most cost-effective and sustainable approach for delivering appropriate malaria treatment services. Also, in order to have an appreciable public health impact in Africa, antimalarials have to be deployed effectively [3].

Management, adequate training, support, and supervision have been recognized as key elements which 'make or break' CHW programmes [10]. When interactions between CHWs and local health personnel are not in harmony, it often negatively impacts such programmes and this may happen when local health personnel are not involved in the set up of the community-based health programme [10]. Hence, in this study, local public health policy makers and malaria control managers should be fully involved in the project from inception to evaluation. The study team also took a long time to train the CHWs, provided continuous logistic and advisory support during the implementation and strictly supervised their work with regular weekly meetings and site visits. People interested in starting similar programmes in their localities should bear in mind that in order to ensure sustainability of such programmes, continued CHWs training can sometimes serve as motivation for CHWs in the absence of remuneration for the services they provide in the community [10]. For instance, in the Philippines, inadequate training, insufficient logistic support, poorly sustained motivational schemes and lack of community support, resulted in below optimal contribution from some CHWs [13].

## Conclusion

As has been shown in this exploratory study, CHWs could be an acceptable and highly preferred means of providing timely and appropriate malaria treatment services. The combined CHW models 3 and 4 [5] worked well in the study context. The CHWs should be seen as a highly effective complementary activity that enhances the value of existing services [10]. The CHWs could be made part and parcel of the primary healthcare (PHC) system akin to Chinese foot doctors [10]. The CHWs should also be paid salaries and provided with training and tools to be able to diagnose malaria using treatment guidelines and rapid diagnostic tests. CHWs have "shown that they can effect major changes in mortality and other indices of health status, and that in certain communities they can satisfy prominent health care needs which cannot realistically be met by other means" [32].

The CHW strategy could be used to improve and ensure near, timely and appropriate treatment of malaria. The requirements now are further studies in different contexts and on larger scales to determine the replicability of these results and to fine-tune and scale up the use of the CHWs strategy in rural parts of Nigeria and even sub-Saharan Africa. Also, an additional body of evidence is needed to show that the strategy works could support the effective implementation of the malaria home-management strategy. Health systems need to provide the CHWs with medications and other supplies, regular supervision and links to a referral system [33]. Hence, it would also be good to fashion how the CHWs strategy could become part of the primary health care system for its sustainability and enhanced status, in addition to symbiotically improving the effectiveness of the public health care system in malaria control.

## Authors' contributions

OO conceived the study, and wrote the first draft of the paper. All the authors participated in data collection, data analysis and reviewing the drafts of the paper, until the final draft was produced.
